# Impaired pressure natriuresis and non‐dipping blood pressure in rats with early type 1 diabetes mellitus

**DOI:** 10.1113/JP277332

**Published:** 2018-12-23

**Authors:** Geoffrey J. Culshaw, Hannah M. Costello, David Binnie, Kevin R. Stewart, Alicja Czopek, Neeraj Dhaun, Patrick W. F. Hadoke, David J. Webb, Matthew A. Bailey

**Affiliations:** ^1^ The British Heart Foundation Centre for Cardiovascular Science The Queen's Medical Research Institute The University of Edinburgh 47 Little France Crescent Edinburgh EH16 4TJ UK

**Keywords:** hypertension, blood pressure, pressure natriuresis, experimental type 1 diabetes mellitus, sodium homeostasis, lithium clearance

## Abstract

**Key points:**

- Type 1 diabetes mellitus increases cardiovascular risk; hypertension amplifies this risk, while pressure natriuresis regulates long‐term blood pressure.
- We induced type 1 diabetes in rats by streptozotocin injection and demonstrated a substantial impairment of pressure natriuresis: acute increases in blood pressure did not increase renal medullary blood flow, tubular sodium reabsorption was not downregulated, and proximal tubule sodium reabsorption, measured by lithium clearance, was unaffected.
- Insulin reduced blood glucose in diabetic rats, and rescued the pressure natriuresis response without influencing lithium clearance, but did not restore medullary blood flow.
- Radiotelemetry showed that diastolic blood pressure was increased in diabetic rats, and its diurnal variation was reduced.
- Increases in medullary blood flow and decreases in distal tubule sodium reabsorption that offset acute rises in BP are impaired in early type 1 diabetes, and this impairment could be a target for preventing hypertension in type 1 diabetes.

**Abstract:**

Type 1 diabetes mellitus (T1DM) substantially increases cardiovascular risk, and hypertension amplifies this risk. Blood pressure (BP) and body sodium homeostasis are linked. T1DM patients have increased total exchangeable sodium, correlating directly with BP. Pressure natriuresis is an important physiological regulator of BP. We hypothesised that pressure natriuresis would be impaired, and BP increased, in the early phase of T1DM. Male Sprague‐Dawley rats were injected with streptozotocin (30–45 mg/kg) or citrate vehicle. After 3 weeks, pressure natriuresis was induced by serial arterial ligation. In non‐diabetic controls, this increased fractional excretion of sodium from ∼1% to ∼25% of the filtered load (*P* < 0.01); in T1DM rats, the response was significantly blunted, peaking at only ∼3% (*P* < 0.01). Mechanistically, normal lithium clearance suggested that distal tubule sodium reabsorption was not downregulated with increased BP in T1DM rats. The pressure dependence of renal medullary perfusion, considered a key factor in the integrated response, was abolished. Insulin therapy rescued the natriuretic response in diabetic rats, restoring normal downregulation of tubular sodium reabsorption when BP was increased. However, the pressure dependence of medullary perfusion was not restored, suggesting persistent vascular dysfunction despite glycaemic control. Radiotelemetry showed that T1DM did not affect systolic BP, but mean diastolic BP was ∼5 mmHg higher than in non‐diabetic controls (*P* < 0.01), and normal diurnal variation was reduced. In conclusion, functional impairment of renal sodium and BP homeostasis is an early manifestation of T1DM, preceding hypertension and nephropathy. Early intervention to restore pressure natriuresis in T1DM may complement reductions in cardiovascular risk achieved with glycaemic control.

## Key points

- Type 1 diabetes mellitus increases cardiovascular risk; hypertension amplifies this risk, while pressure natriuresis regulates long‐term blood pressure.
- We induced type 1 diabetes in rats by streptozotocin injection and demonstrated a substantial impairment of pressure natriuresis: acute increases in blood pressure did not increase renal medullary blood flow, tubular sodium reabsorption was not downregulated, and proximal tubule sodium reabsorption, measured by lithium clearance, was unaffected.
- Insulin reduced blood glucose in diabetic rats, and rescued the pressure natriuresis response without influencing lithium clearance, but did not restore medullary blood flow.
- Radiotelemetry showed that diastolic blood pressure was increased in diabetic rats, and its diurnal variation was reduced.
- Increases in medullary blood flow and decreases in distal tubule sodium reabsorption that offset acute rises in BP are impaired in early type 1 diabetes, and this impairment could be a target for preventing hypertension in type 1 diabetes.

## Introduction

Type 1 diabetes mellitus (T1DM), the common form of diabetes in children and adolescents, increases cardiovascular risk ∼5‐fold (Rawshani *et al*. 2017). Hypertension and albuminuria, more prevalent in T1DM patients than the general population, are major risk factors in this increased burden of cardiovascular disease (de Ferranti *et al*. 2014) and are interrelated: high blood pressure (BP) accelerates the development of diabetic nephropathy (Conway *et al*. 2012); and nephropathy impairs the ability of the kidney to stabilise BP. Hypertension treatment reduces the risk of nephropathy (Shankar *et al*. 2007) and improves cardiovascular outcome in T1DM (Rawshani *et al*. 2017).

The kidneys play an important role in long‐term BP homeostasis, regulating the effective circulating volume through the excretion of sodium chloride. There is a strong positive relationship between arterial pressure, renal perfusion pressure and sodium excretion, termed the pressure natriuresis (PN) response (Ivy & Bailey, 2014). Thus, an acute increase in BP evokes a corresponding increase in blood flow through the renal medullary vasa recta, increasing renal interstitial hydrostatic pressure, which, in turn, inhibits sodium reabsorption in the renal tubule through a combination of physical factors (Starling forces) and paracrine signalling (Ivy & Bailey, 2014). Natriuresis predominantly reflects reduced reabsorption in the proximal tubule, following functional inactivation of major sodium transport proteins. Thus, the sodium‐hydrogen exchanger, NHE3, is redistributed to the base of microvilli in the apical brush‐border membrane and inactivated (Brasen *et al*. 2014); the sodium‐phosphate cotransporter, NaPi2, is removed from the brush border and internalized in endosomes (Riquier *et al*. 2009). Such a significant reduction in proximal tubule reabsorption increases sodium delivery to downstream nephron segments, which normally stimulate the reabsorption of sodium. However, the PN response is integrated so that paracrine factors, such as nitric oxide (NO; O'Connor & Cowley, 2010) and ATP (Menzies *et al*. 2015), suppress compensatory reabsorption in the distal nephron. Intrinsic renal abnormalities (vascular dysfunction; enhanced tubular reabsorption) and extra‐renal factors (sympathetic over‐activity, non‐modulating renin‐angiotensin‐aldosterone system (RAAS), interstitial inflammation) blunt the PN relationship, as observed in both human and experimental hypertension (Wadei & Textor, 2012; Hall, 2016).

We hypothesised that the PN response would be impaired early in T1DM, manifesting as an increased BP. Direct measurement of the PN relationship did, indeed, reveal substantial suppression of the PN curve in rats with uncomplicated T1DM. Although systolic BP was normal in these rats, diastolic BP was elevated and the normal dipping in diastolic BP during sleep was reduced.

## Methods

### Ethical approval

Experiments were performed in accordance with the UK's Animals (Scientific Procedures) Act under a UK Home Office Project Licence. All protocols were reviewed by the University's Animal Welfare and Ethics Review Board prior to experimentation (357‐LF2‐16 and 381‐LF2‐18), and these experiments conform to the principles and regulations described in *The Journal of Physiology* Editorial (Grundy, 2015).

### Origin and source of animals and husbandry

Adult male Sprague Dawley rats, weighing 250–300 g, were purchased from Charles River UK, and transported to Edinburgh under conditions specified in the UK's Animal Welfare Act, 2006. Rats were maintained on standard chow (0.25% sodium) and water *ad libitum*, and were housed in rooms with a 12 h light cycle (lights 07.00–19.00 h) at 21 ± 1°C and 50% humidity. In total, 108 rats were used in this study. Rats were killed by an ASPA‐defined Schedule 1 method prior to study completion if they developed adverse effects associated with T1DM (*n* = 6), failed to become diabetic (*n* = 1) or if radiotelemetry devices malfunctioned (*n* = 4). Experiments, assays and histological scoring were performed with the operator blind to treatment group.

### Induction of T1DM

T1DM was induced by injection of streptozotocin (STZ; 50 mg/ml in 0.1 M citrate buffer, i.p.; Sigma‐Aldrich, UK). All rats received an initial dose of 30 mg/kg and blood glucose was measured after 48 h using a glucometer (Accu‐Chek Aviva; Roche Diagnostics Limited, Burgess Hill, UK). A blood glucose of >12 mmol/l was required to confirm T1DM; some rats did not reach this threshold and a second injection of 15 mg/kg was then given. Blood glucose was again measured at day 7 and at the end of the experimental procedure to confirm sustained hyperglycaemia. Non‐diabetic control rats received vehicle alone. In one group of T1DM rats, blood glucose was controlled to <12 mmol/l by a slow‐release insulin pellet (LinShin, Toronto, Canada) implanted subcutaneously 7 days after confirmation of T1DM. All experiments were performed 2–3 weeks after the final STZ injection.

### Measurement of PN and renal blood flow

Experiments were performed under non‐recovery anaesthesia (Thiopental; 50 mg/kg i.p.; Archimedes Pharma, Reading, UK). The right jugular vein was cannulated for intravenous infusion of physiological saline (pH 7.4; 1 ml/h/100 g body wt) containing 2% (w:v) bovine serum albumin, to limit extravasation, and FITC‐inulin, for measurement of glomerular filtration rate (GFR). General anaesthesia was also maintained through this line by 20–30 μl injections of 50 mg/ml sodium thiopental. A tracheotomy was performed and the right carotid artery cannulated with p50 polyethylene tubing (Smiths Medical International Ltd, Hythe, UK) pre‐flushed with heparinised saline. The arterial line was used for intermittent blood sampling and otherwise was connected to a calibrated BP transducer and multi‐channel data acquisition system (Powerlab; ADInstruments, Oxford, UK) for real‐time BP measurement.

In the first experiment, after a post‐surgical equilibration period of ∼60 min, baseline BP, urine flow rate, urinary sodium excretion rate and GFR were measured over a 30 min period in T1DM and non‐diabetic control rats (Table 1). PN was then induced by sequential arterial ligation of, first, the coeliac and cranial mesenteric arteries, and, second, the distal aorta, as described (Roman *et al*. 1988; Menzies *et al*. 2013), and, after each step of increased BP, urine was collected for 30 min. For the second and subsequent PN experiments, PN was measured in T1DM and non‐diabetic controls but with an additional insulin‐treated diabetic group (T1DM+insulin; Table 1).

**Table 1:** Number, weight, blood glucose (BG) prior to anaesthesia, and mean blood pressures (BP) of control, diabetic (T1DM) and insulin‐treated (T1DM+insulin) rats, during ligature‐induced acute pressure natriuresis

	Expt 1: Effect of T1DM		Expt 2: Role of insulin			Expt 3: Renal blood flow			Expt 4: Lithium clearance		
PN studies	Control	T1DM	Control	T1DM	T1DM+insulin	Control	T1DM	T1DM+insulin	Control	T1DM	T1DM+insulin
Number	8	7	9	7	8	10	11	5	6	6	7
Weight (g)	360 ± 6	354 ± 7	400 ± 13	**338 ± 19** **	363 ± 9	363 ± 7	**331 ± 8** **	**377 ± 23** *	431 ± 16	**359 ± 11** **	381 ± 8
BG (mmol/l)	4.7 ± 0.6	**27.0 ± 1.6** **	4.8 ± 0.2	**16.8 ± 1.8** **	**9.3 ± 0.6** *	6.1 ± 0.3	**16.7 ± 1.7** **	**7.6 ± 0.5** *	4.8 ± 0.2	**32.4 ± 1.0** **	**11.43 ± 2** *
BP 1 (mmHg)	137 ± 3	136 ± 6	111 ± 2	108 ± 4	110 ± 4	124 ± 4	116 ± 4	122 ± 5	92 ± 5	90 ± 3	97 ± 3
BP 2 (mmHg)	153 ± 4	154 ± 5	124 ± 4	119 ± 4	119 ± 3	136 ± 5	128 ± 4	136 ± 4	95 ± 3	97 ± 7	102 ± 5
BP3 (mmHg)	163 ± 4	162 ± 4	147 ± 6	142 ± 5	152 ± 2	144 ± 4	138 ± 5	146 ± 39	117 ± 3	115 ± 8	127 ± 6

Data are means ± standard error of the mean (SEM), ^**^ *P* < 0.05 compared with controls; ^*^ *P* < 0.05 compared with diabetics. All comparisons made with two‐sample Student's *t* tests or one‐way analysis of variance (ANOVA) with Holm‐Sidak's or Tukey's *post hoc* tests.

In the third PN experiment, left renal artery blood flow was measured during PN by Doppler ultrasound, and cortical and medullary flow by Doppler flux (Stern *et al*. 1979). To do this, a calibrated Doppler ultrasound probe (PR‐probe; Transonic, Ithaca, USA) was placed around the left renal artery and ultrasound gel was used for acoustic coupling. The probe was gently rotated to optimise the signal, as visualised by real‐time pulse‐wave recordings, and then the probe was left in place to record renal artery blood flow (RBF; ml/min). Renal cortical and medullary blood flows were estimated by laser Doppler spectroscopy obtained via two separate probes. A patch probe (MSP100XP; ADInstruments) was connected to the dorsal renal surface using tissue glue (Vetbond; 3M, UK) to obtain readings from the cortex; a needle (MNP110XP; ADInstruments) probe was inserted through the capsule to a depth of ∼5 mm, orientated toward the renal hilus. This positioned the probe in the outer medulla, confirmed at post mortem. This probe was stabilised using a micromanipulator and tissue glue was used to further dampen respiratory motion artefact. Doppler spectroscopy infers red blood cell velocity by the frequency modulation of reflected laser light. This Doppler shift is proportional to cell velocity but since the angle of the capillary and the exact concentration of erythrocytes is unknown, an absolute velocity cannot be determined. Instead, data are presented as flux, measured in arbitrary perfusion units, presented relative to the baseline recording of an individual rat (Stern *et al*. 1979).

During the fourth and final PN experiment, rats were infused with a solution containing 10 mmol/l lithium chloride (replacing sodium chloride) to allow measurement of lithium clearance, which provides an index of proximal tubule sodium and water reabsorption, as described (Thomsen & Shirley, 1997).

### Assessment of renal injury and sodium transporter mRNA abundance

A 24 h urine collection was made from T1DM, T1DM+insulin and non‐diabetic control rats (Table 2) 2 weeks after induction of T1DM. The urine concentration of aldosterone was measured using an in‐house ELISA, the specificity and sensitivity of which has been described (Al‐Dujaili *et al*. 2009). Urine albumin (Microalbumin kit; Olympus Diagnostics, Watford, UK) and creatinine (Alpha Laboratories, Eastleigh, UK) were measured on a Cobas Fara centrifugal analyser (Roche Diagnostics Limited). Rats were then killed, the left kidney was removed, and sections of cortex and medulla snap‐frozen for mRNA extraction. Following perfusion‐fixation (4% paraformaldehyde), the right kidney was paraffin‐embedded, sectioned (5 μm) and stained with Haematoxylin and Eosin (to score glomerulosclerosis; Rodriguez‐Iturbe *et al*. 2005) and the pan‐collagen marker, Picrosirius Red (to score fibrosis). Scoring was performed blinded to group under a 20× objective lens (Conway *et al*. 2012).

**Table 2:** Number, weight and blood glucose (BG) of control and diabetic (T1DM) rats that completed the radiotelemetry (RT) study, and control, T1DM and insulin‐treated diabetic rats used in renal injury (RI) studies

	Start		Finish					
RT studies	Control	T1DM	Control	T1DM	RI studies	Control	T1DM	T1DM+insulin
Number	4	4	4	4	Number	8	8	8
Weight(g)	331 ± 4	**317 ± 3** **	586 ± 11	**477 ± 23** **	Weight(g)	383.8 ± 7.7	359.5 ± 12.4	372.7 ± 13.0
BG(mmol/L)	4.4 ± 0.3	**18.7 ± 1.8** **	5.1 ± 0.3	**19.5 ± 1.1** **	BG(mmol/L)	6.8 ± 0.3	**22.0 ± 3.0** **	**10.5 ± 0.9** *

Data are means ± standard error of the mean (SEM), ^**^ *P* < 0.05 compared with controls; ^*^ *P* < 0.05 compared with diabetics. All comparisons made with two‐sample Student's *t* tests or one‐way analysis of variance (ANOVA) with Tukey's *post hoc* tests.

Quantitative RT‐PCR was used to measure the renal abundance of mRNA for genes associated with renal injury/inflammation (*Havcr1*, *Col1a1*, *Col3a1* and *CD68*) and those encoding major sodium transporters (Na, K‐ATPase α and β subunits, NHE3, SGLT2, NKCC2, NCC and  αENaC). Expression was determined using validated TaqMan probes (ThermoFisher Scientific Inc, Glasgow, UK). Following amplification of cDNA, fluorescence of the FAM probes was compared to reference genes GAPDH and TBP using the change in threshold cycle (ΔC_T_) method (Schmittgen & Livak, 2008).

### BP measurement in conscious rats

Radiotelemetry devices (TA11‐CA P40; Data Sciences International, Hertogenbosch, Netherlands) were implanted into eight male Sprague Dawley rats under anaesthesia (inhalational isoflurane, IsoFlo; Zoetis Animal Health Ltd, Sandwich, UK) using aseptic techniques. The sensor tip was fixed into the abdominal aorta, and, after surgery, rats were placed on an insulated mat in a recovery box heated by warm airflow. Post recovery, rats were monitored for a period of at least one hour, and then transferred to individual cages in the radiotelemetry room. Rats received buprenorphine (0.5 mg/kg s.c.; Buprecare; Animalcare, York, UK) every 12 h for 3 days. Systolic BP (SBP), diastolic BP (DBP) and heart rate were acquired over several days to confirm restoration of normal diurnal rhythms. Devices were then turned off using a strong magnet placed over the skin, and rats were randomly allocated to T1DM or non‐diabetic treatment groups (Table 2). The devices were turned on, and data acquired during the fourth week after the first streptozotocin injection. Data were acquired at 1 kHz over a 1 min period in every hour in T1DM and control rats. Zeitgeber time zero (ZT = 0) was defined as the start of the dark period at local time 7pm. Diurnal dipping was calculated for every rat during every day as the percentage reduction in the mean heart rate, mean SBP and mean DBP in the light period compared to the previous dark period. At the end of the experiment, rats were killed by CO_2_ asphyxiation followed by cervical dislocation.

### Statistical analysis

Data are expressed as means ± standard error of the mean (SEM) and analysed with Minitab 17 (Minitab Ltd, Coventry, UK) and GraphPad Prism 6.0 (GraphPad Software, La Jolla, USA). Non‐normal data (Anderson‐Darling test) were compared following log or square‐root transformation, or using non‐parametric tests. For PN studies, two‐way ANOVA with Holm‐Sidak's (two groups) or Tukey's (three groups) *post hoc* tests confirmed similar baseline MBP and increments (independent variable) between groups. Dependent variables were similarly compared, and interaction between diabetic status and clearance period was calculated. As additional analysis, dependent variables were plotted against BP and regression lines compared by analysis of covariance (ANCOVA) with Tukey's *post hoc* tests (linear) and extra sum of squares *F*‐tests (non‐linear). Correlations between dependent variables used Pearson's (normal) or Spearman's rank (non‐normal) tests. For radiotelemetry and assessment of renal injury, single comparisons between groups were made with two‐sample *t* tests (normal) or Mann Whitney *U* tests (non‐normal), while multiple comparisons employed one‐way ANOVA with Tukey *post hoc* tests (normal) or Kruskal Wallis with Dunn's *post hoc* tests (non‐normal). For all tests, statistical significance was set at *P* < 0.05.

## Results

### T1DM impairs the pressure natriuresis response

**Figure 1:**
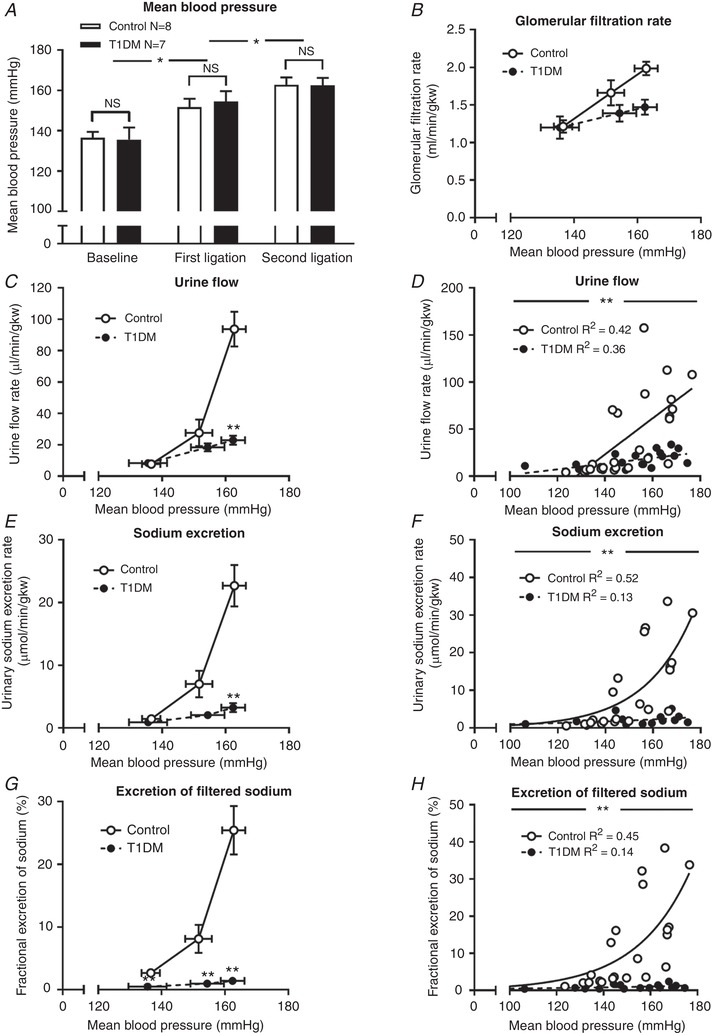
Pressure natriuresis in diabetic and non‐diabetic control rats *A*, baseline mean blood pressure (BP) and increments following arterial ligation in diabetic (T1DM, black bars) and non‐diabetic controls (open bars). Columns show means ± standard error of the mean (SEM), ^*^ *P* < 0.05 compared with mean BP in previous period; NS, not significant. *B*–*H*, the relationships between BP and glomerular filtration rate (*B*), urine flow rate (*C* and *D*), urinary sodium excretion rate (all indexed to kidney weight (kw); *E* and *F*) and fractional excretion of sodium (*G* and *H*) in T1DM rats (black circles) and non‐diabetic control rats (open circles). Data are means ± SEM. ^**^ *P* < 0.01 compared with controls All comparisons made with two‐way analysis of variance (ANOVA) with Holm‐Sidak's *post hoc* tests. Regression lines compared by analysis of covariance (ANCOVA) with Tukey's *post hoc* tests (linear) and extra sum of squares *F*‐tests (non‐linear). *R* ^2^, coefficient of determination. Main effects are described in the text.

Serial arterial ligation was used to impose an acute increase in BP and induce the PN response in non‐diabetic control rats (Table 1). Arterial ligation increased BP by ∼25 mmHg (Fig. 1 *A*), and significantly increased GFR (Fig. 1 *B*; *P* < 0.01) and, therefore, the filtered sodium load. However, the robust diuresis (Fig. 1 *C* and *D*; *P* < 0.01) and natriuresis (Fig. 1 *E* and *F*; *P* < 0.01) that were induced were accompanied by a rise in fractional excretion of sodium (FENa) from ∼1% at baseline to ∼25% at peak perfusion pressure (Fig. 1 *G* and *H*; *P* < 0.01), indicating a substantial inhibition of tubular sodium reabsorption during PN. After 3 weeks of diabetes, rats in the T1DM group had similar BP at baseline and during each of the pressure‐ramps (Fig. 1 *A*) but the renal response was very different from non‐diabetic control animals. The maximum absolute diuresis (Fig. 1 *C* and *D*) and natriuresis (Fig. 1 *E* and *F*) were suppressed by ∼80% (both *P* < 0.01). The increase in GFR was also blunted, but only by ∼0.2 ml/min/g kidney wt (gkw) (Fig. 1 *B*; *P* = 0.01). FENa was significantly lower at baseline in T1DM rats at ∼0.5% (*P* < 0.01) and increased to only ∼3% at peak perfusion pressure (Fig. 1 *G* and *H*).

### Effect of insulin treatment on the pressure natriuresis response

**Figure 2:**
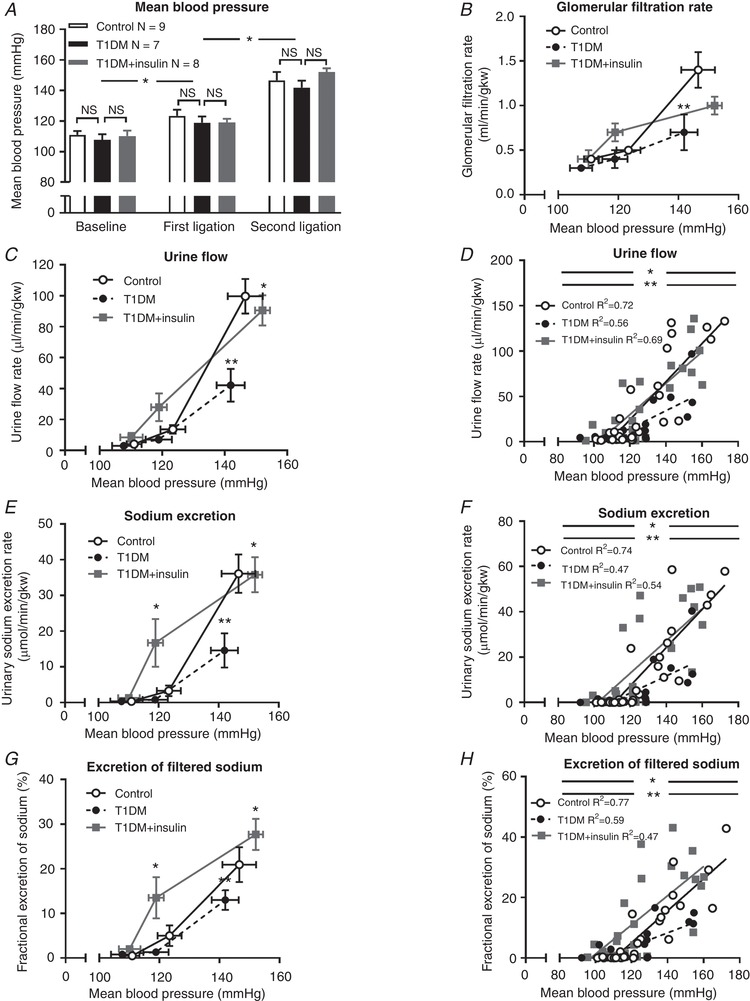
Pressure natriuresis in control, diabetic (T1DM) and insulin‐treated diabetic (T1DM+insulin) rats *A*, baseline mean blood pressure (BP) and increments following arterial ligation in diabetic (T1DM, black bars), insulin‐treated diabetic rats (T1DM+insulin, grey bars) and non‐diabetic control rats (open bars). Columns show means ± standard error of the mean (SEM), ^*^ *P* < 0.05 compared with mean BP in previous period; NS, not significant. *B*–*H*, the relationships between BP and glomerular filtration rate (*B*), urine flow rate (*C* and *D*), urinary sodium excretion rate (all indexed to kidney weight; *E* and *F*) and fractional excretion of sodium (*G* and *H*) in T1DM rats (black circles), insulin‐treated T1DM+insulin rats (grey squares) and non‐diabetic control rats (open circles). Data are means ± SEM. ^**^ *P* < 0.01 compared with controls; ^*^ *P* < 0.01 compared with T1DM. All comparisons made with two‐way analysis of variance (ANOVA) with Tukey's *post hoc* tests. Regression lines compared by analysis of covariance (ANCOVA) with Tukey's *post hoc* tests. *R* ^2^, coefficient of determination. Main effects are described in the text.

PN was next measured in three groups of rats: non‐diabetic controls, T1DM, and insulin‐treated T1DM. Insulin therapy significantly reduced plasma glucose compared to untreated T1DM rats, although it remained higher than in non‐diabetic control animals (Table 1). Baseline BP did not differ between the three groups, and sequential arterial ligation again induced significant increments, totalling ∼35 mmHg, that did not differ between groups (Fig. 2 *A*). GFR (Fig. 2 *B*), urine flow (Fig. 2 *C* and *D*), sodium excretion (Fig. 2 *E* and *F*), and FE_Na_ (Fig. 2 *G* and *H*) increased significantly with BP in all three groups, and the maximum responses were again suppressed in T1DM rats, by up to 60% (all *P* ≤ 0.02). Chronic insulin treatment of diabetic rats effectively restored these relationships to control levels. Indeed, the slopes of the pressure diuresis (Fig. 2 *D*) and PN (Fig. 2 *F*) responses were not significantly different between non‐diabetic control and insulin‐treated diabetic rats. By contrast, in the untreated T1DM group, the slopes of these relationships were, in each case, significantly blunted (*P* < 0.01; Fig. 2 *D* and *F*), despite GFR being unaffected by diabetic status (Fig. 2 *B*), suggesting that impaired PN reflects diabetes, rather than an off‐target effect of STZ.

### Mechanism of impaired PN in T1DM: renal haemodynamics

**Figure 3:**
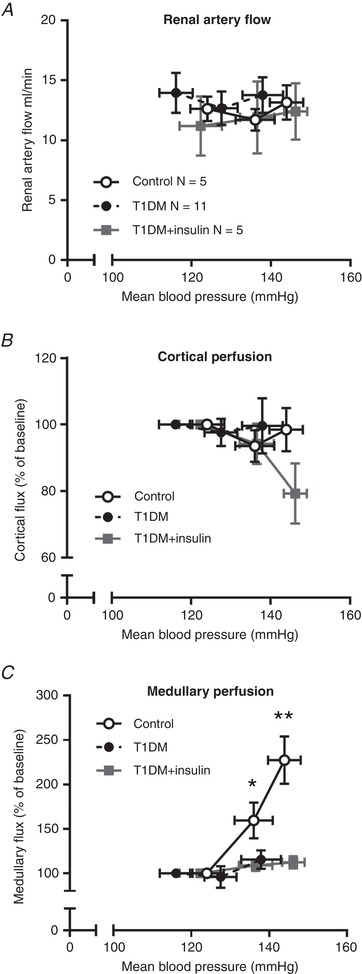
The relationship between arterial pressure and renal haemodynamics The relationship between arterial blood pressure and left renal artery blood flow (*A*), perfusion of the renal cortex (*B*) and perfusion of the renal medulla (*C*) in diabetic rats (T1DM, black circles), insulin‐treated diabetic rats (T1DM+insulin, grey squares) and non‐diabetic control rats (open circles). Data are means ± standard error of the mean (SEM). ^**^ *P* < 0.01 compared with T1DM only; ^*^ *P* < 0.01 compared with either T1DM or T1DM+insulin, using two‐way analysis of variance (ANOVA) and Tukey's *post hoc* tests. Main effects are described in the text

RBF and perfusion of the renal cortex and medulla, were measured at baseline and during sequential arterial ligation (Table 1). RBF (Fig. 3 *A*) was not different between groups at baseline and did not change significantly with increased BP, suggesting effective autoregulation. Perfusion of the cortex and medulla was measured by Doppler flux and, for group comparison, baseline recordings were normalised to 100%. Cortical flux did not change with increased BP in non‐diabetic rats, falling slightly, but not significantly in T1DM and insulin‐treated groups at the highest perfusion pressure (Fig. 3 *B*). In non‐diabetic control rats, perfusion of the medulla increased significantly with arterial pressure (Fig. 3 *C*). However, this pressure dependence of medullary flux was not observed in T1DM rats. Importantly, insulin therapy, which had restored the natriuretic response to increased BP (Fig. 2 *E* and *F*), did not restore the pressure dependence of medullary blood flow in T1DM rats (Fig. 3 *C*).

### Mechanism of impaired PN in T1DM: tubular sodium reabsorption

**Figure 4:**
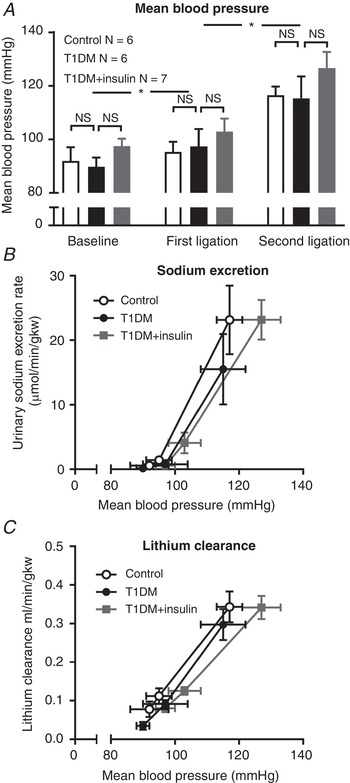
Lithium clearance in control, diabetic and insulin‐treated diabetic rats *A*, baseline mean blood pressure (BP) and increments following arterial ligation in diabetic (T1DM, black bars), insulin‐treated diabetic rats (T1DM+insulin, grey bars) and non‐diabetic control rats (open bars). Columns show means ± standard error of the mean (SEM), ^*^ *P* < 0.05 compared with mean BP in previous period; NS, not significant. *B* and *C*, the relationships between BP and urinary sodium excretion rate (*B*) and lithium excretion (*C*) (both indexed to kidney weight) in T1DM rats (black circles), insulin‐treated T1DM+insulin rats (grey squares) and non‐diabetic control rats (open circles). Data are means ± SEM. All comparisons made with two‐way analysis of variance (ANOVA) with Tukey's *post hoc* tests. Main effects are described in the text.

In the final PN experiment, we determined the effect of increasing BP on the renal lithium clearance (*C*_Li_) in additional rats in the same three groups (Table 1). As before, sequential arterial ligation significantly increased BP in all three groups of rats (*P* < 0.01), here by an average of ∼25 mmHg (Fig. 4 *A*). Sodium excretion increased with BP (*P* < 0.01) and was again lower in T1DM rats than in the other two groups, but, with peak BP ∼10–20 mmHg lower than in the previous PN experiments, this did not reach statistical significance (Fig. 4 *B*). At baseline BP, neither T1DM (0.04 ± 0.01 ml/min/gkw) nor T1DM+insulin (0.08 ± 0.01 ml/min/gkw) affected *C*_Li_ (control, 0.08 ± 0.01 ml/min/gkw), while increasing BP led to a similar significant increase in *C*_Li_ in all three groups (Fig. 4 *C*; *P* < 0.01).

### T1DM did not induce renal injury or changes in major sodium transporter expression

Renal injury was assessed at the 3‐week time point. In T1DM rats, the urinary albumin:creatinine ratio was ∼1.6‐fold greater than that of non‐diabetic control rats (*P* = 0.02) but we could not detect glomerulosclerosis on histopathological assessment. Picrosirius Red staining was used to report collagen abundance and this did not differ between groups in either the renal cortex (T1DM 0.52 ± 0.08% *vs*. control 0.41 ± 0.02%) or medulla (T1DM 0.29 ± 0.03% *vs*. control 0.36 ± 0.03%). Transcriptional markers that are sensitive to renal injury, *Havcr*1, *Col1a1*, *Col4a1* and *CD68*, were measured in the renal cortex and medulla and were not different between groups. We found no change in the mRNA abundance of the major renal sodium transporter proteins (Na,K‐ATPase α and β subunits, NHE3, SGLT2, NKCC2, NCC and  αENaC), and the urinary:aldosterone creatinine ratio, indicative of RAAS activity, was not different between groups (data not shown).

### T1DM increases diastolic blood pressure and impairs dipping

**Figure 5:**
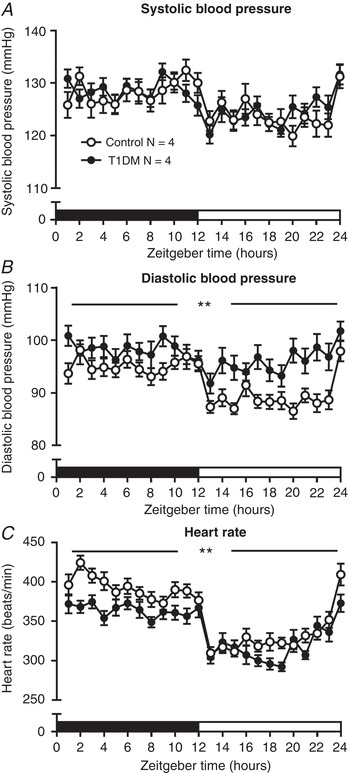
Blood pressure and heart rate Blood pressure and heart rate were measured by radiotelemetry every hour over 5 days in diabetic (T1DM) rats (black circles) and non‐diabetic control rats (open circles). Zeitgeber time zero is the start of the dark period (black bar), the open bar is the light period. Data points are group means ± standard error of the mean (SEM) over a 24 h cycle. *A*, systolic blood pressure; *B*, diastolic blood pressure; *C*, heart rate. ^**^ *P* < 0.05 for mean value over 24 h in diabetic rats compared with controls, using two‐sample Student's *t* test or Mann Whitney *U* test according to normality of data. Main effects are described in the text.

Finally, we determined whether the impaired PN response in uncomplicated T1DM was associated with an increased BP. Radiotelemetry probes were implanted into T1DM and non‐diabetic rats (Table 2), permitting longitudinal recording of SBP, DBP and heart rate in conscious, unrestrained animals. After 3 weeks of diabetes, SBP (Fig. 5 *A*) was comparable to control animals and also displayed normal diurnal variation (Table 3), as assessed by both calculating the dip in SBP from dark (active) to light (sleep) periods, and by using cosinor analysis to generate mesor (as the central tendency), the amplitude of the diurnal variation and the acrophase of the 24 h cycle.

**Table 3:** Means, dips and cosinor analysis values for blood pressure and heart rate

	SBP		DBP		Heart rate	
	Control	T1DM	Control	T1DM	Control	T1DM
Mean (mmHg/bpm)	126.2 ± 0.5	126.5 ± 0.4	92.2 ± 0.4	**97.1 ± 0.4** **	361.7 ± 2.5	**341.5 ± 2.3** **
Dip (%)	3 ± 1	3 ± 1	6 ± 1	**2 ± 1** **	15 ± 1	12 ± 1
Mesor (mmHg/bpm)	127.4 ± 2.9	126.5 ± 1.1	91.4 ± 1.2	94.0 ± 2.7	362.3 ± 11.1	336.7 ± 17.5
Amp (mmHg/bpm)	2.9 ± 0.5	3.1 ± 0.3	4.1 ± 0.4	**1.8 ± 0.7** **	45.8 ± 4.5	35.4 ± 3.8
Acro (radians)	69.3 ± 4.4	71.3 ± 1.7	81.6 ± 2.9	46.2 ± 15.8	71.1 ± 2.6	76.6 ± 6.8

Data were recorded by radiotelemetry in diabetic (T1DM; *n* = 4) rats and non‐diabetic controls (*n* = 4), and are expressed as mean ± standard error of the mean (SEM). ^**^ *P* < 0.05 compared with controls, using two‐sample Student's *t* tests for means and dips, and Welch's *t* test for cosinor analysis. SBP, systolic blood pressure; DBP, diastolic blood pressure; bpm, beats per minute; Amp, amplitude; Acro, acrophase.

By contrast, mean DBP was increased in T1DM rats by ∼5 mmHg (*P* < 0.01), and the normal diurnal variation was suppressed such that the dip in diastolic BP during the light phase was reduced by 4% (Table 3; Fig. 5 *B*; *P* < 0.01), reflected also in a significant reduction in the cyclic amplitude (*P* = 0.04, Table 3). Heart rate was less in T1DM rats by ∼20 beats/min (Table 3; Fig. 5 *C*; *P* < 0.01) but displayed normal diurnal variation in both groups.

## Discussion

PN is the integrated tubulovascular response to increased renal perfusion pressure (Ivy & Bailey, 2014). It is thought to stabilise long‐term BP at a given set‐point (Guyton, 1987), and functional impairment is considered an important early event leading to the development of hypertension (Wadei & Textor, 2012; Hall, 2016). In established hypertension, the PN curve is suppressed/right‐shifted (DeClue *et al*. 1978; Norman *et al*. 1978; Kimura *et al*. 1987). This abnormal BP homeostasis manifests initially as attenuation or loss of the normal nocturnal BP dip (Fukuda *et al*. 2012). Normally, elevated renal arterial perfusion pressure induces a PN response consisting of both haemodynamic and tubular components. Here, we show in anaesthetised rats that uncomplicated T1DM substantially blunts both components: (i) blood flow in the renal medulla is uncoupled from arterial pressure and (ii) sodium transport through the tubule epithelium fails to down‐regulate with increased perfusion pressure. In non‐diabetic rats, the applied pressure ramps increased GFR and the filtered sodium load, elevated blood flow in the renal medulla, and reduced tubular sodium reabsorption. T1DM affected all elements of the PN response: increases in GFR and medullary blood flow were blunted, while tubular sodium reabsorption did not decrease substantially.

We do not consider the lack of pressure‐induced hyperfiltration to be a major factor in the blunted natriuresis observed in T1DM rats: even in healthy rats, GFR does not consistently increase during PN and *in vivo* micropuncture studies show that single nephron GFR is largely autoregulated across the range of pressure used here (Haas *et al*. 1986; Roman *et al*. 1988). Thus, the contribution of increased filtration to the overall natriuretic response is likely to be small, even in the non‐diabetic controls. Similar findings are reported in patients with uncomplicated T1DM. They exhibit a blunted natriuretic response to saline infusion (Roland *et al*. 1986) or water immersion (O'Hare *et al*. 1989), also attributed to avid renal sodium retention, while maintaining or even increasing creatinine clearance, a clinical marker of GFR. Furthermore, we do not believe that structural nephropathy contributed to impaired PN in T1DM rats, since we found no evidence of renal injury by histological, biochemical or sensitive molecular approaches.

Of greater biological significance is the uncoupling of medullary flux from arterial pressure in T1DM rats. Increased blood flow through the vasa recta causes interstitial pressure to rise throughout the kidney (Garcia‐Estan & Roman, 1989) and is widely held to be a key process for PN (Roman *et al*. 1988; O'Connor & Cowley, 2010). The pressure dependence of renal medullary flux was attenuated in T1DM rats. It could be speculated that such vascular dysfunction reflects reduced bioavailability of NO (Pflueger *et al*. 1999; Persson *et al*. 2017), an important mediator of the rise in vasa recta blood flow and the PN response (Lockhart *et al*. 1994). Indeed, T1DM impairs the normal hyperaemic response of the vasa recta to NO (Palm *et al*. 2005; Persson *et al*. 2014) and alterations in both NO production and vascular sensitivity would be expected to uncouple medullary flux from arterial pressure. Such haemodynamic uncoupling has been suggested by earlier experiments in which early T1DM diminished the rise in renal interstitial hydrostatic pressure following an acute saline load (Patel & Carmines, 2001). Surprisingly, we found that restoring normal blood glucose levels with insulin normalised the PN curve without re‐coupling medullary flux to arterial pressure. This unanticipated finding is consistent with the restoration of the natriuretic response to a saline load that occurs in pregnant diabetic rats, despite continued suppression of renal interstitial pressure (Tang *et al*. 2004), and challenges the prevailing view that medullary hyperaemia is an essential component of the PN response.

It is well established that T1DM increases basal tubular sodium reabsorption, as we report here. In terms of molecular mechanism, some studies (Ward *et al*. 2001; Song *et al*. 2003) report increased expression of NHE3 (proximal tubule), NKCC2, (loop of Henle), NCC (distal convoluted tubule) and ENaC (collecting duct) in T1DM rats but this is not a consistent finding (Nejsum *et al*. 2001). In our study, T1DM did not increase the mRNA expression of these sodium transporters, and we therefore utilised *C*_Li_ to localise the tubular defect in T1DM rats. The central tenets of the *C*_Li_ approach are that lithium is reabsorbed in the proximal tubule in direct proportion to sodium and water but is not reabsorbed in the loop of Henle and the distal nephron. Micropuncture studies in rodents indicate that the *C*_Li_ approach is less accurate in some disease settings, including T1DM (Pollock & Field, 1992), but nevertheless it remains a valuable qualitative marker of proximal tubule function (Thomsen & Shirley, 1997). Our data clearly show that *C*_Li_ increases with BP to a similar extent in both T1DM and non‐diabetic rats. Thus, the proximal tubule modulates appropriately in T1DM rats within a mean BP range of ∼90–130 mmHg, and the tubular defect accounting for an impaired PN response in early T1DM is in the distal nephron.

The identification of the causative physiological mechanism is an important step towards defining the underpinning molecular pathways of impaired PN. It is possible that an individual transport system in the loop of Henle, distal convoluted tubule or collecting duct fails to turn off as perfusion pressure rises. Against this hypothesis is the restoration of the natriuretic response by insulin therapy demonstrated here, since insulin activates sodium transporter pathways (Komers *et al*. 2012; Mansley *et al*. 2016). A more compelling speculation is that an inhibitory paracrine pathway fails to respond to increased BP, and distal sodium reabsorption is sustained rather than integrated into the overall natriuretic response. NO, discussed above, is an attractive potential candidate. Another candidate is ATP, which is normally released in response to increased renal perfusion pressure (Palygin *et al*. 2017) and inhibits tubular sodium transport through ENaC (Menzies *et al*. 2015). Notably, genetic deletion of connexin 30 (Sipos *et al*. 2009), which mediates ATP release in the distal tubule (Palygin *et al*. 2017), impairs the PN response in mice.

Studies in patients show that sodium retention occurs early in T1DM, correlating positively with BP (Feldt‐Rasmussen *et al*. 1987). Our data suggest that restoration of PN may help improve long‐term sodium homeostasis in patients with T1DM but the study does not establish a direct and causal relationship between loss of the acute PN response and hypertension. Indeed, whether a causal relationship exists is a current controversy in cardiovascular research. The Guytonian hypothesis remains influential (Hall, 2016) but other authorities, while acknowledging that renal excretory impairment is a *sine qua non* for hypertension, argue for a new paradigm (Evans & Bie, 2016). Indeed, several studies, including from our laboratory (Evans *et al*. 2016), show salt‐sensitive hypertension in the absence of demonstrably impaired renal function, most likely reflecting an abnormal vasodilatory response to effective circulatory volume expansion and increased cardiac output (see Morris *et al*. 2016 for review). Here, we report both vascular and tubular dysfunction in T1DM rats. These animals were not hypertensive *per se* but the mean 24 h DBP was increased by ∼5 mmHg, which could not be explained on the basis of tachycardia, since heart rate was consistently less than in controls. Ambulatory BP monitoring also revealed a reduction in diastolic dipping during the sleep phase of the diurnal cycle. In T1DM patients, reduced BP dipping and an increase in daytime DBP but not SBP, precedes and predicts the onset of albuminuria (Lurbe *et al*. 2002). Non‐modulating sodium transport may be causal such that elevated nocturnal BP is required to facilitate sodium excretion and restore balance (Bankir *et al*. 2008) but at the expense of glomerular exposure to prolonged haemodynamic stress. In a wider context, there is some evidence that therapeutic restoration of BP rhythm reduces cardiovascular risk (Hermida *et al*. 2018) and our work raises the possibility that impairment of PN may be a suitable therapeutic target for reducing cardiovascular risk in T1DM before structural nephropathy develops.

## Conclusion

We conclude that PN is impaired at an early stage of T1DM, prior to the onset of nephropathy and contemporaneous with increased DBP and loss of diastolic dip. We propose that such impairment is an important event in the progression of T1DM, rendering BP homeostasis vulnerable to a ‘second hit’, such as habitually high salt intake (Gray *et al*. 2014). Early intervention to restore the normal relationship between arterial pressure and tubular sodium reabsorption could have long‐term benefits for renal and cardiovascular risk in T1DM.

## Additional information

### Competing interests

The authors declare no competing interests.

### Author contributions

G.J.C., P.W.F.H., D.J.W. and M.A.B. contributed to the conception or design of the work. G.J.C., H.M.C., D.B., K.R.S. and A.C. contributed to the acquisition of data. G.J.C., H.M.C., N.D., P.W.F.H., D.J.W. and M.A.B. analysed or interpreted data. G.J.C., M.A.B. and D.J.W. drafted and revised the manuscript. All authors approved the final manuscript and agree to be accountable for the work. All persons designated as authors qualify for authorship, and all those who qualify for authorship are listed.

### Sources of funding

This work was supported by a Kidney Research UK Project grant (RP2/2014), British Heart Foundation (BHF) Centre of Research Excellence (CoRE) Awards (RE/08/001/23904; RE/13/3/30183), The Roslin Institute, and a BHF Intermediate Clinical Research Fellowship to N.D. (FS/13/30/29994). Travel awards to present preliminary data were awarded by the BHF CoRE and The Physiological Society.

**Translational perspective**

We hypothesised that the pressure natriuresis response (PN) is impaired early in type 1 diabetes mellitus (T1DM), manifesting as an increased blood pressure (BP). Our data demonstrate that there is a severe, hyperglycaemia‐dependent impairment of PN in early T1DM that is normalised by insulin, and occurs before the development of structural nephropathy. It is not associated with systolic hypertension, but, instead, with an increased mean diastolic BP and decreased diastolic dipping of BP that are analogous to the increase in daytime diastolic BP observed in T1DM patients prior to nephropathy (Lurbe *et al*. 2002). These findings are consistent with, first, reductions in cardiovascular risk observed with tight blood glucose control in clinical T1DM (Nathan *et al*., Diabetes Control and Complications Trial/Epidemiology of Diabetes Interventions and Complications Study Research Group, 2005), and, second, changes in BP that predict nephropathy in T1DM (Lurbe *et al*. 2002). Clinically, tight blood glucose control achieves maximal reductions in cardiovascular risk within a crucial early window after diagnosis of T1DM (Nathan *et al*., Diabetes Control and Complications Trial/Epidemiology of Diabetes Interventions and Complications Study Research Group, 2005). However, as T1DM is most frequently diagnosed in young children, this approach increases the risk of life‐threatening hypoglycaemia. We have identified impaired PN as a potential target for cardiovascular protection in early T1DM that may complement insulin therapy, especially where tight blood glucose control is not achieved or practicable.
